# Low Expression of hsa_circ_0018069 in Human Bladder Cancer and Its Clinical Significance

**DOI:** 10.1155/2019/9681863

**Published:** 2019-03-10

**Authors:** Mingshan Li, Yixiang Wang, Yili Liu, Xiling Zhang, Jie Liu, Ping Wang

**Affiliations:** ^1^Fourth Affiliated Hospital of China Medical University, Shenyang 110032, China; ^2^Science Experiment Center of China Medical University, Shenyang 110122, China

## Abstract

Abnormal expression of noncoding RNA molecules such as circRNA plays an important role in the development of malignant tumors. circRNAs are stable in structure and can be useful as ideal tumor markers. Advanced bladder cancer has poor treatment options and prognosis. Thus, we examined circRNAs to further understand the pathogenesis and development of bladder cancer and to identify molecular markers for the early diagnosis of bladder carcinoma. We found that hsa_circ_0018069 was differentially expressed in our RNA sequencing data. We used qRT-PCR to detect its expression in T24 and Biu-87 cell lines and in 41 paired samples of bladder cancer and adjacent normal tissue and analyzed the correlation between expression of hsa_circ_0018069 and the clinical characteristics of patients with bladder cancer. We then performed a bioinformatics analysis to reveal the mechanism of hsa_circ_0018069 in tumorigenesis of bladder cancer. The expression of hsa_circ_0018069 was significantly reduced in T24 and Biu-87 cells and was also significantly downregulated in bladder cancer tissues. Decreased expression of hsa_circ_0018069 was related to the grade stage (P=0.024), T stage (P=0.027), and muscular invasion depth (P=0.022) of bladder cancer. Bioinformatics analysis showed that hsa_circ_0018069 was coexpressed with protein-coding mRNAs that participate in cytoskeletal protein binding and cell-substrate junction assembly and play an anticancer role through focal adhesion and calcium signaling pathways. ceRNA analysis showed that hsa_circ_0018069 functions in ErbB, Ras, FoxO, and the focal adhesion signaling pathway by harboring miR-23c, miR-34a-5p, miR-181b-5p, miR-454-3p, and miR-3666. hsa_circ_0018069 may thus play an important role in the occurrence and progression of bladder cancer and serve as a valuable biomarker for the early diagnosis of this disease.

## 1. Introduction

Bladder cancer is one of the most common tumors in the urinary system [1]. Surgical operation is the main treatment for bladder cancer. However, when tumors advance to a high grade, the result of treatment is frequently unsatisfactory. Patients with high-risk bladder cancer may relapse, progress or die within 10 years [2]. The 5-year survival rate of high grade muscular invasive bladder cancer is only 6% [3]. Advanced bladder cancer displays poor sensitivity to radiotherapy and chemotherapy; therefore, early diagnosis of bladder cancer is particularly important.

In recent years, next-generation sequencing technology has been employed for transcriptome analysis and has provided valuable information on the diagnosis and underlying mechanisms of malignant carcinoma. With this technology, many noncoding RNAs have been discovered and studied, which has shown that abnormal expression of circRNA plays an important role in the formation of bladder tumors [4]. In our previous study [5], we found hundreds of circRNAs were up- or downregulated in bladder cancer tissues compared with adjacent tissues, of which hsa_circ_0018069 was one of the most significantly downregulated circRNAs both confirmed by sequencing and real-time-PCR. It is spliced from chr10:30315031-30318795 with a length of 3764 nt and is formed by circularization of the fourth exon of* KIAA1462*. In the present study, we show that hsa_circ_0018069 plays an important role in bladder cancer and may be useful as a biomarker for early diagnosis.

## 2. Results

### 2.1. Differentially Expressed Transcripts of circRNAs

**Figure 1:**
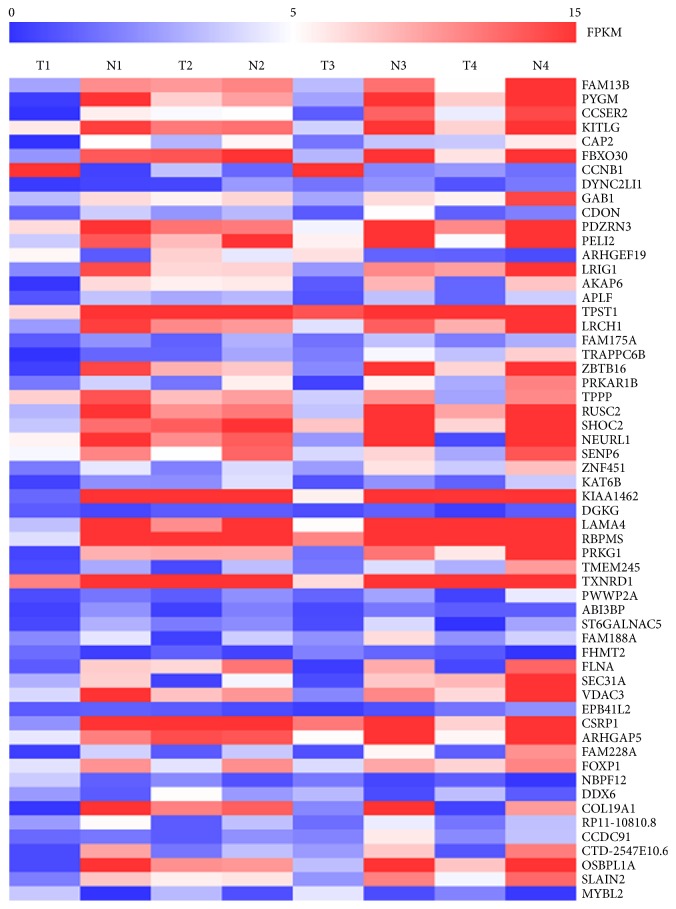
Heatmap of significant differentially expressed transcripts of circRNAs. Blue color designates low expression; red color designates high expression.

We performed RNA sequencing on four out of 41 pairs of bladder cancer tissue. In total, 59 differentially expressed transcripts of circRNAs were observed in the bladder cancer tissues with a fold-change ≥2.0,* P*<0.05 and q<0.05. Compared with noncancerous adjacent tissues, seven circRNAs were upregulated while 52 circRNAs were downregulated in bladder cancer tissues. The significant differences in circRNA transcript expression between tumors and adjacent tissues are represented in a heatmap (Figure 1).

### 2.2. Expression of hsa_circ_0018069 in Cells and Tissues

**Figure 2:**
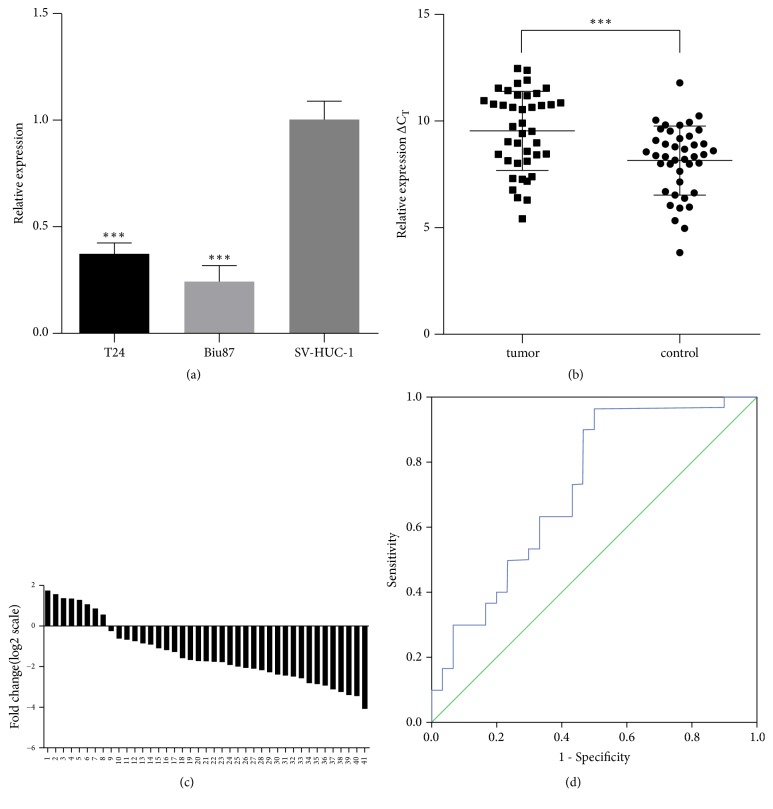
(a) Relative expression of hsa_circ_018069 in bladder cancer cell lines (T24 and Biu87) and the normal bladder epithelial cell line SV-HUC-1. Data are expressed as the means ± SD, and asterisks indicate significant P-values (*∗∗∗*P<0.001). (b) hsa_circ_018069 expression levels in 41 pairs of bladder cancer and adjacent tissues. (c) The expression level of hsa_circ_018069 was significantly downregulated in 80.5% (33/41) of bladder cancer tissues compared with adjacent normal tissues. (d) ROC curve.

Our sequencing results showed that hsa_circ_0018069 was downregulated in bladder cancer (Table 1). We performed qRT-PCR in the cell lines Biu-87, T24, and SV-HUC-1; the results showed that hsa_circ_0018069 was downregulated 4.24- and 2.69-fold, respectively, compared with SV-HUC-1(Figure 2(a)). We also examined in 41 pairs of cancerous and normal adjacent tissues and found that hsa_circ_0018069 expression was downregulated in most bladder cancer tissues (P<0.05; Figures 2(b) and 2(c)). In order to assess the diagnostic value of hsa_circ_0018069, we plotted a ROC curve. The area under the curve was 0.709 (Figure 2(d)) and the cutoff value was 0.0007, with sensitivity of 0.976 and specificity of 0.463.

### 2.3. Expression Level and Clinical Relevance of hsa_circ_0018069

Based on the above data, we analyzed the correlation between the expression level of hsa_circ_0018069 and clinical data of cancer patients. As shown in Table 2, downregulation of hsa_circ_0018069 was significantly associated with grade stage (P=0.024), T stage (P=0.027) and muscular invasion depth (P=0.022), but was not associated with age, sex, tumor size or lymphatic metastasis.

### 2.4. Bioinformatics Analysis

**Figure 3:**
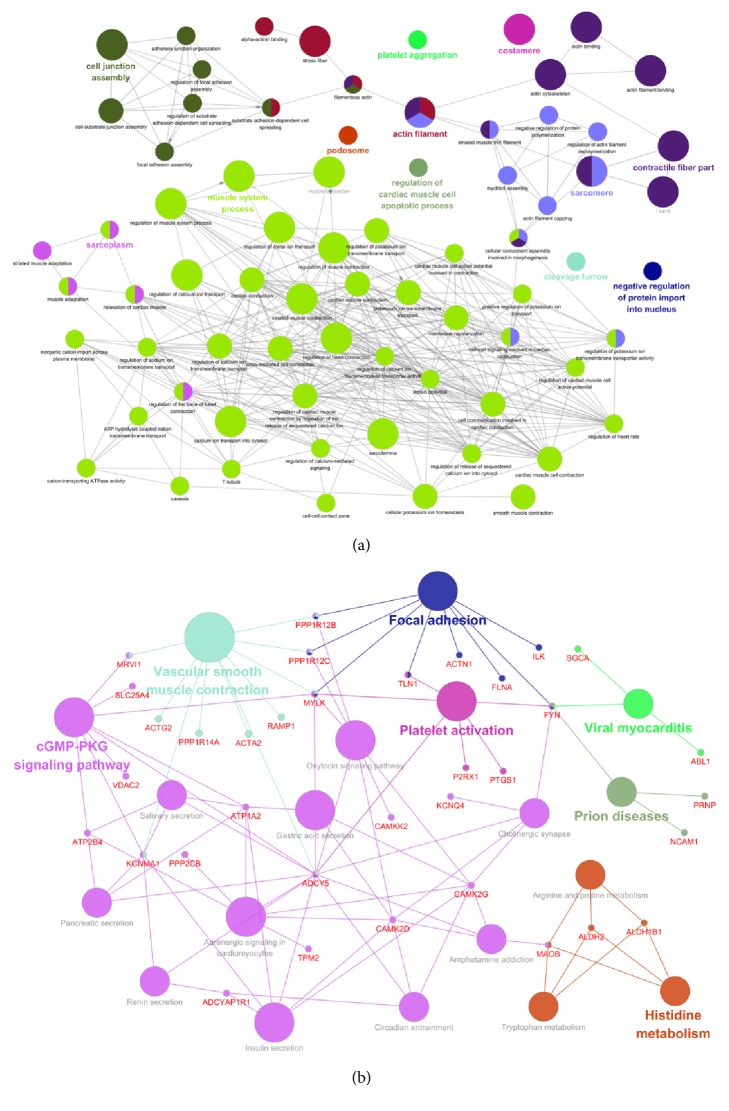
(a) GO analysis for coexpressed mRNAs of hsa_circ_018069 with a relationship >0.95 or <-0.95. (b) KEGG analysis for coexpressed mRNAs of hsa_circ_018069. Bubble scale represents kappa scores of enriched terms.

**Figure 4:**
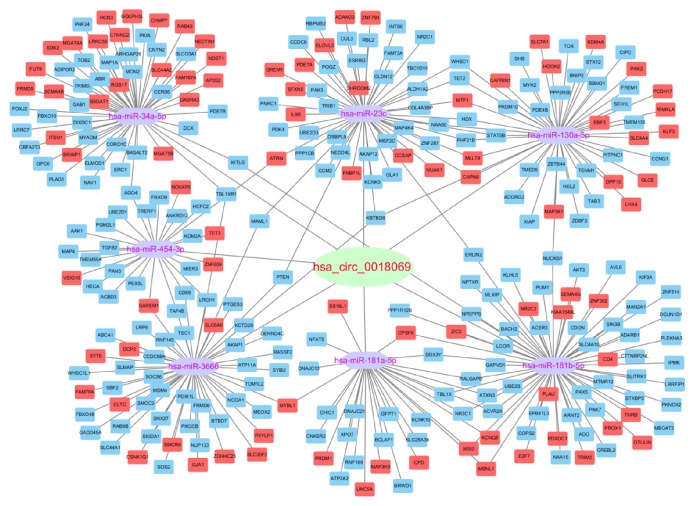
ceRNA analysis for hsa_circ_018069. Red nodes represent upregulated mRNA; blue nodes represent downregulated mRNA.

**Figure 5:**
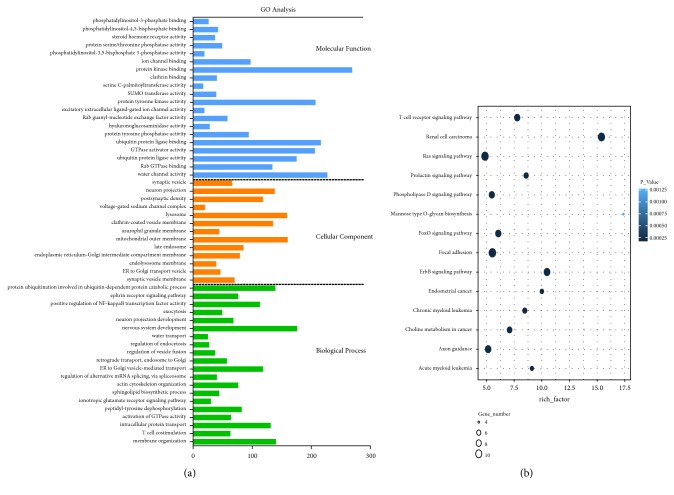
(a) GO analysis for mRNAs participating as ceRNAs of hsa_circ_018069. (b) KEGG analysis for mRNAs participating as ceRNAs of hsa_circ_018069. Enrichment factor represents the ratio between the differentially expressed gene and all annotated genes enriched in the pathway. Bubble scale represents the number of different genes; depth of bubble color represents P value.

We calculated Pearson's correlation coefficients among hsa_circ_0018069 and differentially expressed mRNAs and selected genes with positive correlations >0.95 and negative correlations <-0.95 for further study. We performed GO (Figure 3(a)) and KEGG (Figure 3(b)) analysis on coexpressed mRNAs with a group P value <0.05. Cytoskeletal protein binding and actin binding were the significant enriched GO terms, and focal adhesion and cGMP-PKG signaling were the main enriched pathways. We predicted miRNAs that could be sponged by hsa_circ_0018069 and predicted targeted mRNAs based on the ceRNA mechanism. The mapped ceRNA network showed that hsa_circ_0018069 could target* AKT3, SOS1, PIK3CB, PTEN, FOXO3, DIXDC1,* and* PPP1R12B* by sponging miR-23c, miR-34a-5p, miR-181b-5p, miR-454-3p, and miR-3666 (Figure 4). We also performed GO and KEGG analysis for the targeted mRNAs (Figures 5(a) and 5(b)) and showed that hsa_circ_0018069 may play a tumor suppressor role through the ErbB, Ras, FoxO, and focal adhesion signaling pathways.

## 3. Discussion

Noncoding RNA was once considered meaningless for transcription, but recent studies have shown that it plays a vital role in the progression of disease [6]. CircRNA, without a 5' end cap and a 3' end poly-A tail, has a ring structure formed by covalent bonds and is resistant to exonuclease [7, 8]. It is currently believed that circRNA is formed through a reverse splicing mechanism [9]. CircRNA can act as ceRNA, which is involved in the regulation of gene expression. It can also bind to proteins in the nucleus and modulate the function of transcription factors. Certain circRNAs have internal ribosome entry sites and may encode proteins. Low expression of hsa_circ_002059 was confirmed to be related to overexpression of CEA and distant metastasis in gastric cancer [10], demonstrating that circRNA may contribute to clinical diagnosis and prognosis of tumors. However, the function of most circRNAs is not clear. In our sequencing data, we found that hsa_circ_0018069 was significantly downregulated in four pairs of tumor and adjacent tissues, and we also examined the expression of hsa_circ_0018069 in bladder cell lines and tissues by qRT-PCR. Results showed that in the T24 and Biu87 cell lines and in 33 out of 41 pairs of tissues, hsa_circ_0018069 was downregulated, which indicates that hsa_circ_0018069 plays an anticancer role in the occurrence and development bladder cancer.

Currently, most functions of circRNAs have been predicted by RNA-binding proteins or ceRNA mechanisms. In addition, we can also predict the function of circRNAs through correlated mRNAs. However, we found no RNA-protein binding sites in hsa_circ_0018069 by online software. We therefore performed a coexpression and ceRNA analysis to elucidate the function of hsa_circ_0018069. GO analysis of coexpressed mRNAs showed that the main enriched pathways were the focal adhesion and cGMP-PKG signaling pathways. The focal adhesion pathway was reported to be associated with embryonic development, tumor development and migration [11]. The genes enriched in the pathway include* ACTN1, FLNA, FYN, ILK, MYLK, PPP1R12B, PPP1R12C,* and* TLN1*.* FLNA* is associated with cell proliferation and invasion in hepatocellular carcinoma [12], while* TLN1* participates in the invasion of ovarian serous carcinoma and is active in hepatocellular carcinoma cells [13, 14]. We therefore deduced that hsa_circ_0018069 may be involved in invasion of bladder cancer through the focal adhesion pathway. Another major enriched pathway was cGMP-PKG signaling, which plays an anticancer role in colon cancer [15] and renal cancer [16]. These two enriched pathways revealed that hsa_circ_0018069 may act as an antioncogene in bladder cancer.

ceRNA analysis showed that hsa_circ_0018069 could sponge miR-23c, miR-34a-5p, miR-181b-5p, miR-454-3p, and miR-3666. KEGG analysis of mRNAs showed enrichment in the ErbB, Ras, FoxO, and focal adhesion signaling pathways. In various kinds of malignant tumors, ErbB family members and partial ligands are frequently overexpressed, amplified, or mutated. For example, research revealed that* EGFR* was amplified and/or mutated in glioma and non-small-cell lung cancer [17], whereas* ErbB2* was amplified in breast, ovarian and bladder cancer and in several other tumors [18, 19]. ErbB receptor signaling regulates cell proliferation, migration, differentiation, apoptosis, and cell mobility in Akt, MAPK, and other pathways [20–23]. The Foxo signaling pathway is closely related to the insulin/PI3K/AKT signaling pathway and interacts with tumor suppressor gene* P53* [24, 25]. FOXO signaling plays an inhibitory role in ovarian cancer, prostate cancer and colorectal cancer by inducing cell cycle arrest and apoptosis. In our study, hsa_circ_0018069 was downregulated in bladder cancer tissues and exerts an anticancer effect, which suggested that hsa_circ_0018069 may mediate the Foxo signaling pathway. Focal adhesion was a common pathway enriched in both KEGG pathway analysis of coexpressed mRNAs and in ceRNA analysis. The shared mRNA was* PPP1R12B*, which was significantly downregulated in bladder cancer in our sequencing result and in the TCGA database [26]. From our bioinformatics analysis, we speculate hsa_circ_0018069 is involved in pathways related to tumorigenesis and development of bladder cancer mainly through sponging miR-181b-5p to suppress expression of* PPP1R12B*.

The diagnosis of bladder cancer is mainly confirmed based on the patient's symptoms, signs and clinical examination. Hematuria is a common symptom, but the incidence of gross hematuria in bladder cancer only accounts for 17% to 18.9% [27, 28]. Ultrasound is valuable in diagnosis of bladder cancer, and transurethral bladder ultrasound shows high accuracy in tumor staging but is useless in the diagnosis of carcinoma in situ. Accuracy of diagnosis by CT and urine cytology is slightly lower, and interfering factors often lead to misdiagnosis. Currently, there is still no ideal biomarker that can replace cystoscope and urine cytology detection for bladder cancer and improve diagnosis, treatment, prognosis and postoperative follow-up [29]. Our research indicated that the accuracy of hsa_circ_0018069 in the diagnosis of bladder cancer can reach 97.6%, with specificity of 46.3% and an AUC of 0.709. This indicated that hsa_circ_0018069 may be valuable in the diagnosis of bladder cancer. The expression of hsa_circ_0018069 was also correlated with T stage and muscular invasion, revealing that hsa_circ_0018069 may serve as a preliminary marker to estimate tumor invasion. Combined with our bioinformatics analysis, we conclude that hsa_circ_0018069 plays a vital role in the invasion of bladder cancer. The expression of hsa_circ_0018069 can be combined with bladder microscopy results to determine clinical staging. Follow-up detection of hsa_circ_0018069 can then be obtained from peripheral blood, which can reduce biopsy damage to patients and provide a new approach for the early diagnosis of bladder cancer.

## 4. Conclusion

In our study, hsa_circ_0018069 was significantly downregulated and affected the T stage and invasion of bladder cancer. Our analysis suggested that hsa_circ_0018069 played a significant role in the development of bladder cancer and may be useful as a biomarker for the early diagnosis of this disease.

## 5. Materials and Methods

### 5.1. Specimen and Clinical Data Collection

From October 2016 to October 2017, a total of 41 pairs of bladder cancer tissues and adjacent tissues were collected from the Department of Urology of the Fourth Affiliated Hospital of China Medical University (Shenyang, China). The adjacent tissues were more than 3 cm away from the tumor tissues. All patients included in the study were initially diagnosed without any treatments and were subjected to total or partial cystectomy. Diagnosis was confirmed by postoperative pathology. Bladder cancer was classified according to the World Health Organization international classification of oncology and TNM staging of the Union for International Cancer Control [30]. The experiment was approved by the Ethics Committee of China Medical University and all patients signed informed consent.

### 5.2. Sequencing Process and Analysis

Whole transcriptome sequencing was performed on four out of 41 pairs of bladder cancer tissues and adjacent tissues using the HiSeq X instrument. CIRCexplorer [31] was used to predict circRNAs. A criterion of |log2(fold-change)|≥ 1, P value <0.05 and q <0.05 between two samples was used to identify differentially expressed genes and transcripts.

### 5.3. Cell Culture

Bladder cell lines T24, Biu-87, and normal uroepithelial SV-HUC-1 cells were purchased from the Shanghai Cell Bank of Chinese Academy of Sciences (Shanghai, China). T24 and Biu-87 cells were cultivated with RPMI 1640 (Gibco, MA, USA) and 10% fetal bovine serum (Hyclone, MA, USA). SV-HUC-1 cells were cultivated with F12K (Gibco, MA, USA) and 10% fetal bovine serum. All cells were cultivated in a 37°C incubator with 5% CO_2_.

### 5.4. Extraction of Total RNA and qRT-PCR

TRIzol (Thermo Fisher, MA, USA) was used to extract RNA from tissues. Quantitative real-time PCR was used to detect the expression of hsa_circ_0018069. The primer sequences for hsa_circ_0018069 were 5'-GAGCATCCGGCAGCACAAAA-3' (forward), 3'-TCCGGTAGGCTTGGTCGTTA-5' (reverse). The primer sequences for the *β*-actin internal reference were 5'-GAGACCTTCAACACCCCAGCC-3' (forward), 3'-GGATCTTCATGAGGTAGTCAG-5' (reverse). All primers were synthesized by Shanghai Shenggong Company (Shanghai, China). The PCR reaction conditions were set at 95°C for 5 s and 60°C for 34 s in a total of 40 cycles. The data was analyzed by ΔCT.

### 5.5. Bioinformatic Analysis

The target miRNAs for hsa_circ_0018069 and target genes for those miRNAs were determined with online software miRWalk3.0 (http://129.206.7.150/), Targetscan (http://www.targetscan.org/), and mirDB (http://www.mirdb.org/). RBP sites were predicted by circinteractome (https://circinteractome.nia.nih.gov/). The correlation between the hsa_circ_0018069 and differentially expressed genes was analyzed using Pearson's correlation coefficient. GO and KEGG analysis were performed for coexpressed and targeted genes, and the ceRNA network was mapped by Cytoscape 3.6 (http://www.cytoscape.org).

### 5.6. Statistical Analysis

Statistical analysis was performed with Student's* t*-test using SPSS 22.0 (IBM, Armonk, NY, USA) and P-values <0.05 were considered statistically significant. GraphPad Prism 6 (GraphPad, San Diego, CA, USA) and MeV4 (http://mev.tm4.org) were used to draw the figures.

## Figures and Tables

**Table 1:** Transcript expression levels of hsa_circ_018069 from sequencing results.

Sample	Tumor(FPKM)	Control(FPKM)	log2(fold_change)
1	1.29572	41.4469	-4.999438304
2	20.2475	19.8868	0.025932637
3	5.71587	33.413	-2.547364512
4	14.979	44.1508	-1.559498267

**Table 2:** The association of hsa_circ_018069 expression levels with clinicopathological characteristics of bladder cancer patients.

*Characteristics*	*No of Cases*	*Mean±SD∗*	P value
Age(years)			
≥60	33	1.33±1.64	0.749
<60	8	1.53±1.21	
Sex			
Male	36	1.40±1.57	0.760
Female	5	1.17±1.58	
Grade			
G1	8	0.27±1.41	
G2	-	-	*0.024*
G3	33	1.64±1.48	
Diameter(cm)			
≥5cm	10	1.88±0.42	0.156
<5	31	1.10±0.28	
T stage			
T1	9	0.35±1.33	
T2	16	1.29±1.69	*0.027*
T3	16	2.03±1.23	
T4	-	-	
Clinical stage			
1	9	0.35±1.33	0.071
2	14	1.26±1.81	
3	14	2.03±1.32	
4	4	1.80±0.39	
Lymphatic metastasis			
N1-2	4	1.80±0.39	0.164
N0	37	1.33±1.62	
Invasion depth			
NMIBC	10	0.35±1.33	*0.022*
MIBC	31	1.66±1.50	

*∗* Mean±SD was present in CT

## Data Availability

The data used to support the findings of this study are available from the corresponding author upon request.

## Abbreviations

circRNA: Circular RNA
miRNA: MicroRNA
ceRNA: Competing endogenous RNAs
FPKM: Reads Per Kilobase of exon model per Million mapped reads
CT: Cycle threshold
AUC: Area under the curve
ROC: Receiver operating characteristic
NMIBC: Non-Muscle Invasive Bladder Cancer
MIBC: Muscle Invasive Bladder Cancer
GO: Gene ontology
KEGG: Kyoto Encyclopedia of Genes and Genomes
RBP: RNA-binding protein.
